# The *E. coli* CNF1 as a Pioneering Therapy for the Central Nervous System Diseases

**DOI:** 10.3390/toxins6010270

**Published:** 2014-01-07

**Authors:** Sara Travaglione, Stefano Loizzo, Giulia Ballan, Carla Fiorentini, Alessia Fabbri

**Affiliations:** Department of Therapeutic Research and Medicines Evaluation, Superior Health Institute, viale Regina Elena 299, Rome 00161, Italy; E-Mails: sara.travaglione@iss.it (S.T.); stefano.loizzo@iss.it (S.L.); giukkab@gmail.com (G.B.); carla.fiorentini@iss.it (C.F.)

**Keywords:** CNF1, actin cytoskeleton, synaptic plasticity, Rho GTPases

## Abstract

The Cytotoxic Necrotizing Factor 1 (CNF1), a protein toxin from pathogenic *E. coli*, modulates the Rho GTPases, thus, directing the organization of the actin cytoskeleton. In the nervous system, the Rho GTPases play a key role in several processes, controlling the morphogenesis of dendritic spines and synaptic plasticity in brain tissues. This review is focused on the peculiar property of CNF1 to enhance brain plasticity in *in vivo* animal models of central nervous system (CNS) diseases, and on its possible application in therapy.

## 1. Introduction

*Escherichia coli* is a normal inhabitant of the large bowel that becomes highly pathogenic upon acquisition of genes encoding virulence factors. Pathogenic strains of *E. coli* are involved in both intestinal and extra-intestinal infections. The Cytotoxic Necrotizing Factor 1 (CNF1) is one of the virulence factors mainly produced by extraintestinal *E. coli*, in particular those correlated to neonatal meningitis [1], bacteriaemia [2], and urinary tract infections (UTIs) [3,4,5,6]. Although the pathogenic role of CNF1 is not fully clarified, most reports evidence the ability of the toxin to induce “beneficial effects” in cells, at least in term of acquisition of new cellular abilities [7,8,9,10,11,12,13]. One of the most intriguing “new abilities” promoted by CNF1 is the polymerization of the actin cytoskeleton into stress fibers and membrane ruffling, which is due to the activation of the regulatory small G-proteins belonging to the Rho family [14,15,16]. The ability of Rho proteins to modulate the organization of the actin network drives many aspects of cell behavior, including morphogenesis, migration, phagocytosis, and cytokinesis [17]. In the nervous system, the Rho GTPases, via the actin cytoskeleton, play a key role in several processes, such as the morphogenesis of the dendritic spines of neurons in the brain [18,19,20] and synaptic plasticity [21,22,23,24,25,26]. It is interesting to note that dysregulation of Rho GTPases is associated with numerous human diseases and disorders and is causative of some forms of intellectual disability [27]. All this is in agreement with the recently described role of CNF1 in the CNS where CNF1 exerts a positive role mainly consisting in the improvement of neuronal plasticity [28] and in the amelioration of the behavioral phenotype in certain neurological and neurodevelopmental diseases [29,30]. In this context, CNF1 can be view as a promising pharmacological compound derived from the microbial world. Some other bacterial toxins have already been studied and employed as supply for therapeutic applications. One of the best characterized and the first recognized as a potential drug in a broad range of neurological pathologies is the botulinum neurotoxin. This toxin, also known as an efficient instrument for the reduction of facial wrinkles [31], has been used since the 1970s for strabismus, blepharospasms, focal dystonias, and in the treatment of disorders characterized by excessive or inappropriate muscle contraction, such as spasticity, eye movement disorders, hyperhidrosis, sphincter spasms, or hemifacial spasms (for a review see [32]). Moreover, scientific and clinical evidence has emerged that suggests multiple antinociceptive mechanisms for botulinum toxins in a variety of painful disorders (reviewed in [32]).

Few other bacterial toxins have been described to have therapeutic properties, such as the antrax toxin, which represents a novel and potentially useful chemotherapeutic [33,34], or the pertussis toxin that can block the HIV infectious process *in vitro* at multiple levels [35,36]. However, for these last toxins, preclinical studies are taking place to better understand their mechanism of action.

## 2. About CNF1

### 2.1. The Molecular Point of View

CNF1 is a single-chain multidomain protein that contains a N-terminal receptor-binding domain, a translocation domain (middle domain) and a C-terminal catalytic domain, which modifies a specific cellular target in the host cell cytosol [37,38]. It binds to the surface of cultured cells with high affinity [39] through both the ubiquitously expressed 37 kDa precursor of the 67 kDa laminin receptor (LRP) [40], and the mature laminin receptor [41]. Moreover, competition studies with CNF1 suggest that heparan sulfate proteoglycan (HSPG) is a co-receptor for the toxin since inhibition of HSPG expression causes delayed cell entry of CNF1 [42]. 

After binding to its receptor, CNF1 is internalized by endocytosis and subsequently transferred to an endosomal compartment by a microtubule dependent mechanism [39]. At this level, conformational changes, resulting from the acidification of late endosomes, drive the translocation of the enzymatic domain into the cytoplasm where CNF1 is cleaved in an approximately 55-kDa fragment that is necessary for full biological activity of the toxin [43]. It was postulated that the hydrophilic loop, which separates H1 and H2 helices present in the middle part of the CNF1 molecule, is essential for translocation of the toxin into the host cell cytosol [44].

The cytoplasmic target of CNF1 is represented by the Rho GTP-binding proteins, important molecular switches of the Ras superfamily that oscillate between an inactive GDP-bound form and an active GTP-bound one which activates effector proteins. Transitions between the two forms are primarily under the control of activators (guanine nucleotide exchange factors, GEFs) and inactivators (GTPase-activating proteins, GAPs) [45]. The enzymatic activity of the toxin consists in the deamidation of a specific glutamine residue, located in the switch 2 domain of the small G proteins (glutamine 63 of Rho [14,15] or glutamine 61 of Rac and Cdc42 [16]), and essential for the GTPase activity of Rho proteins [46]. By modifying glutamine into glutamic acid, CNF1 allows Rho proteins to be permanently locked in their activated GTP-bound state and, thus, enhances the activity of these proteins on their effectors.

The threshold of activation of Rho proteins by CNF1 is, however, attenuated because of a concomitant decrease of their cellular levels, due to the depletion of activated-Rho GTPases by proteasomal degradation [47]. To be addressed to proteasome, proteins must be first ubiquitylated through a complex molecular mechanism that involves a cascade of transfer reactions between ubiquitin-carrier proteins [48]. In this process, the ubiquitin-ligase E3 plays a pivotal role, conferring substrate specificity to the reaction [48,49]. It is worth noting that CNF1 activates Rho GTPases in all cell systems where its effects have been studied, but the timing and level of activation of distinct members of Rho family differ in the different cell types [47,50], depending on the efficiency of their ubiquitylination apparatus. As a consequence, CNF1 can be considered not simply an activator of Rho GTPases, but, rather, a Rho modulator.

### 2.2. CNF1 and Eukaryotic Cells

Rho GTPases regulate the organization and dynamic of the actin cytoskeleton through their interplay with specific effector proteins [45,51,52]. Indeed, by activating Rho proteins, CNF1 provokes a remarkable reorganization of the actin cytoskeleton in human epithelial cells that consists in the assembly of F-actin in prominent stress fibers, membrane ruffles, and filopodia [53,54]. In addition to assuming a well-spread phenotype, CNF1-treated cultured cells also undergo multinucleation, a phenomenon that probably arises from an ongoing nuclear division accompained by a cytoskeleton-dependent inhibition of cytokinesis [7].

The extensive modifications of the actin microfilament system induced by the toxin provoke a potent and aspecific phagocytic-like behavior, epithelial cells acquiring the ability to capture and internalize different types of particles, such latex beads, apoptotic cells, and also bacteria, through an endocytic mechanism known as macropinocytosis [7,10]. As above described, CNF1-activated Rho GTPases undergo sensitization to ubiquitylation and subsequent proteosomal degradation, a process that would turn off the ruffling process, thus, allowing an efficient internalization of bacteria inside the cells. As degradation of Rac increases cell motility and leads to enhanced internalization of bacteria, it was speculated that sequential Rho GTPase activation and inactivation limits the inflammatory response and promotes survival of bacteria [47].

In addition to regulating the actin cytoskeleton organization Rho, Rac, and Cdc42 are also involved in many other cellular processes. Thus, the consequence of CNF1-induced Rho activation is the induction of a number of actin-dependent or -independent phenomena, such as contractility, cell spreading [53], assembly of focal adhesion plaques [55], transcription [11], cell cycle regulation [13], as well as the ability to manipulate cell differentiation [12].

As concerns DNA transcription, we reported that CNF1 can activate the nuclear factor-kB (NF-kB) in epithelial cells [11]. Such an activation is responsible for the ability of the toxin to stimulate the expression of pro-inflammatory factors [56] and to protect host cell from apoptotic stimuli [8,9,57]. Hence, CNF1 is not cytotoxic, as suggested by its name, but rather can be considered as a pro-survival factor. Indeed, we have shown that CNF1 hinders UVB-induced apoptotic cell death in a carcinoma cell line. In particular, the toxin protects cells from the experimentally-induced rounding up and detachment and improves cell spreading and the ability of cells to adhere to each other and to the extracellular matrix by modulating the expression of proteins related to cell adhesion (integrins, focal adhesion kinase, cadherins, catenins) [8]. Moreover, CNF1 protects epithelial cells against the drop of the mitochondrial membrane potential provoked by UVB radiation and increases the expression of the anti-apoptotic proteins Bcl-2 and Bcl-XL [9] through the activation of the Rac1/PI3K/Akt/IKK/NF-kB pro-survival pathway [57]. This, in turn, leads to a remarkable modification in the architecture of the mitochondrial network, mainly consisting in the appearance of elongated and interconnected mitochondria, which contributes to the pro-survival activity of the toxin [57].

## 3. CNF1 on Neuronal Cells and Its Promising Therapeutic Effects

In the nervous system, the Rho GTPases play a key role in several processes, and mutations in proteins involved in Rho GTPase signaling may be causative of some forms of intellectual disabilities. In fact, the ability of Rho GTPases to modulate the organization of the actin network plays important roles in the morphogenesis of the dendritic spines of neurons in the brain and in the synaptic plasticity [58]. 

**Figure 1:**
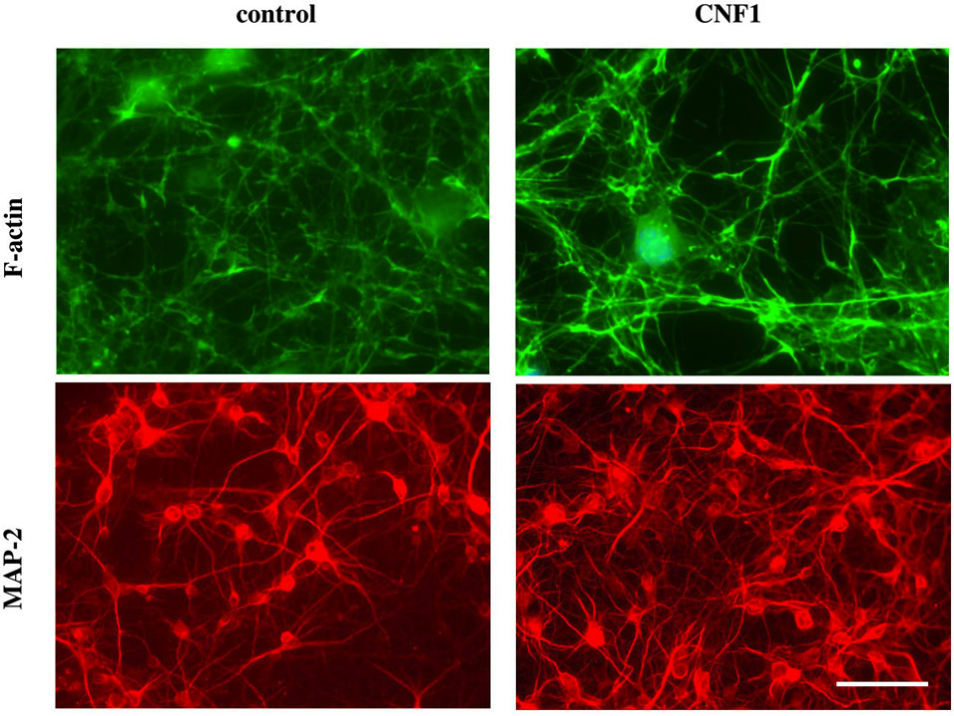
Astrocytes challenged with CNF1 provide a more efficient substrate to neuronal growth. F-actin (upper panels) and MAP-2 (lower panels) staining of hippocampal neurons growing on CNF1-treated astrocytes. To note that hippocampal neurons, growing on CNF1-treated astrocytes, produce a much more abundant dendritic tree, with a richer branching, creating a confluent network. Bar represents 50 μm.

Very recently, we have analyzed the effects of CNF1 on primary neuronal and astrocytic cultures [28], discovering that, under the toxin influence, astrocytes increase their supporting activity on neuronal growth and differentiation. Indeed, CNF1 profoundly affects differentiation of pure hippocampal neurons, decreasing synapse formation and inducing the formation of thick and tortuous dendrites devoid of ramifications. By contrast, hippocampal neurons, growing on CNF1-treated astrocytes, but in absence of direct CNF1 influence, produce a much more abundant dendritic tree, with a richer branching, creating a confluent network, as shown by actin and MAP-2 immunolabeling (Figure 1). Furthermore, the enlargement of the dendritic tree is accompanied by an increased formation of synapses. The production of interleukin 1b (IL-1b), as well as the content of fibrillary acidic protein (GFAP), were decreased in CNF1-exposed astrocytes [28]. Accordingly, it is known that IL-1b reduces dendrite development and complexity in neuronal cultures [59], and GFAP reduction has been put in relation to increased astrocytic-induced dendritogenesis [60]. The beneficial effect of CNF1 on neuronal cells may be at the basis of a very promising therapeutic activity of the toxin as highlighted, in recent years, in several preclinical studies. 

### 3.1. Improvement of the Analgesic Activity

The first report dealing wih CNF1 as a putative drug dates back to 2009. In this context, we have reported that peripheral or central administration of CNF1 potently counteract formalin-induced inflammatory pain in mice [61]. Inflammatory pain is controlled by a complex framework of molecular events, either at the cell surface or intracellularly, which have only partially been elucidated. In particular, it seems that the actin cytoskeleton influences pain-related signaling [62,63,64]. Formalin injection induced inflammatory pain and evoked licking behavior, which had the characteristic biphasic temporal trend: the early short-lasting phase (phase 1), due to the direct stimulation of nociceptors and initiation of inflammation, and the second prolonged phase (phase 2) reflecting pain-sensitization processes [65]. We have shown that the two phases (phases 1 and 2), characterizing formalin-induced pain, are affected by CNF1 in different ways, depending on the route of administration. In fact, only intracerebroventricular (icv) injection of the toxin is able to affect both phases, indicating a central modulating action of CNF1 on neural processes associated with both the acute phase and the inflammatory tonic phase of the formalin-induced pain. By contrast, peripherally injected CNF1 influenced only the second phase. The analgesic response due to CNF1 required both the sustained activation of the Rac GTPase, with consequent cerebral actin cytoskeleton remodeling, and the up-regulation of the m-opioid receptors (MORs), the most important receptors controlling pain perception. We can hypothesize that the higher availability of MORs promoted by the toxin could provide additional links to the endogenous opioids released after formalin stimulation, thus, reducing the pain threshold.

### 3.2. CNF1 and Functional Plasticity in the Adult Rat Visual Cortex

As described above, Rho GTPases represent key regulators of the shape and morphological plasticity of axons, dendrites, and dendritic spines [66]. In this context, we have reported that CNF1 is able to increase brain plasticity in adulthood in a classical model of age-dependent plasticity, the ocular dominance (OD) plasticity in rats, triggered by monocular deprivation (MD) [67]. Sensitivity of cortical circuits to a brief period of MD is maximal in juvenile animals and downregulated in adult age [68]. The injection of CNF1 into the primary visual cortex of naive adult rats (older than 90 post-natal days), triggered a long-lasting activation of the Rho GTPase Rac1, with a consequent increase in spine density and length in pyramidal neurons. Adult rats treated with CNF1, but not controls, showed an OD shift toward the open eye after MD. CNF1-mediated OD plasticity is selectively attributable to the enhancement of open-eye responses, whereas closed-eye inputs are unaffected. This effect correlates with an increased density of geniculocortical terminals in layer IV of monocularly deprived, CNF1-treated rats. Thus, Rho GTPase activation reinstates OD plasticity in the adult cortex via the potentiation of more active inputs from the open eye [67]. These data establish a direct link between structural remodeling and functional plasticity and demonstrate a role for Rho GTPases in brain plasticity *in vivo*. The ability of CNF1 to act on synaptic plasticity by Rho GTPases activity modulation may be exploited to promote brain repair.

### 3.3. CNF1 and Rett Syndrome

In line with their central role in controlling structural plasticity and actin cytoskeleton dynamics [45,69], aberrant Rho signaling has been reported to be associated with abnormalities in dendrites and spines in non-syndromic intellectual disability, and to be responsible for cognitive impairments [27,70]. In particular, Rho GTPases signaling pathways have been suggested to be involved in the pathophysiology of a clinical variant of Rett syndrome (RTT) [71], a rare and severe neurodevelopmental disorder and a genetic cause of intellectual disability. 

In our previous works, we showed that CNF1 (i) is able to enhance learning and memory performances; (ii) induces a re-arrangement of cerebral actin cytoskeleton; and (iii) enhances neurotransmission and synaptic plasticity in animal models. All these effects persisted for weeks and were strictly dependent on Rho GTPases activation [61,72,73]. To assess whether pharmacological interventions targeting Rho GTPases may be an effective therapeutic strategy for RTT, we evaluated the effects of a single icv injection of CNF1 in MeCP2-308 hemizygous (hz) male mice, a mouse model of RTT that expresses a truncated form of the MeCP2 gene [74,75,76,77]. We have demonstrated that CNF1 administration markedly improves the behavioral phenotype of MeCP2-308 mice, and dramatically reverses the evident signs of atrophy in astrocytes of mutant mice, restoring the morphology of astrocytes and the values of GFAP-positive areas (the astrocytic marker) close to the wild-type levels. Moreover, CNF1 buffered the intracellular energy levels in mutant mice, as confirmed by increased phosphocreatine content in both hippocampal and striatal areas [29]. A partial rescue of the overexpression of IL-6 cytokine was also observed in RTT brains, this last result being in line with the finding that CNF1 reduces the expression of pro-inflammatory cytokines, such as IL-6, in astrocytes *in vitro* [28]. This study provides the first evidence of the potential efficacy of a new treatment for the RTT syndrome, based on modulation of Rho GTPases, and indicates that the beneficial effects on several RTT-related behavioral domains of CNF1 are mediated by a restoration of astrocytic function and involve mitochondria functionality.

### 3.4. CNF1 and Alzheimer’s Disease

Deficits in neuronal plasticity have been reported not only in Rett syndrome, but also in several other pathologies of the CNS, including Alzheimer’disease (AD). AD is characterized by an increment of neuroinflammatory markers, such as pro-inflammatory cytokines and b-amyloid, and by energy and cognitive deficiencies [78]. Very recently, we have investigated the effects of CNF1 on transgenic mice homozygous for the human isoforme variant apoE4, a validated sporadic AD [79,80] and atheroschlerosis [81] murine model, characterized by a rapid age-related cognitive decline associated with neuroinflammatory responses [82,83], mitochondrial dysfunction and accumulation of b-amyloid in the brain [84,85,86]. 

Also in this case, a single icv injection of CNF1 is effective in inducing an improvement of the performances of the mouse model used. In particular, CNF1 induces a long lasting strong amelioration of both spatial and emotional memory deficits, favors the cell energy restore through an increment of ATP content and decreases the levels of b-amyloid accumulation and interleukin-1b expression in the hippocampus of aged (one year old) apoE4 mice [30]. All these effects are accompanied by a modulation of cerebral Rho and Rac1 activity. The striking improvement of the cognitive defects in CNF1-treated mice is most probably linked, via the pharmacological modulation of Rho GTPase signaling, to a restoration of physiological energy levels and to anti-inflammatory processes, endorsing CNF1 as a potential therapy against AD, atherosclerosis, and neuroinflammation diseases in general. 

## 4. Conclusions

This review has been focused on CNF1 and on its peculiar property to improve dendritic spine density and neurotransmission functionality, thus, enhancing brain plasticity. These findings pave the way to a possible pharmacological approach to those CNS diseases that lack so far of an efficient neuroprotective or disease-modifying therapy. 

We can assume that following injection into the ventricle, CNF1 first reaches, through the cerebral blood flux, the astrocytic compartment and that the activity of the toxin on these cells is at the basis of the observed *in vivo* effects [28,29,30]. As, in non-transformed cells, the effect of CNF1 on the Rho GTPases are lowered by proteasomal degradation, it is reasonable to conceive that the long-term effects observed in CNF1-treated mice are due to the persistence of the molecular effects induced by the toxin rather than to the persistence of the toxin itself in the CNS. In fact, CNF1 has profound effects on the cytoskeleton, on proteins’ expression and distribution, and on mitochondrial activity that persist for long periods of time in mouse brain [29,30]. It is interesting to note that known modulators of Rho GTPases capable of passing the blood brain barrier have been already used in preclinical studies, indicating the potential benefit of Rho pathway inhibition. Among the others, we can mention Fasudil and Y-27632 (for a review see [87]), two classes of widely used chemical compounds that inhibit Rho kinase (ROCK), an important downstream effector of RhoA subfamily GTPases. On the other hand, compounds directly targeting Rho GTPases are only represented, to our knowledge, by the Rac1 inhibitor NSC23766 that acts on ischemic models increasing cognitive performances [88]. In this context, despite the invasive route of CNF1 administration so far employed, yet the proved ability of CNF1 to revert, after a single injection, cognitive impairment in fully symptomatic pathological CNS animal models represents an invaluable advantage. Furthermore, the observed benefits are long-lasting. 

Therefore, CNF1 could be viewed in the future as one of those natural products at the basis of drugs used in the most varied diseases. We are aware that the microbial world is apparently a very unlike source for therapeutics, yet bacteria, with their toxins, can disclose new possibilities that deserve investigation.
